# Identifying Clinician Perspectives on Current Stroke Care Gaps Across the Care Continuum: An Ontario, Canada-Focused Study

**DOI:** 10.1177/11786329261486701

**Published:** 2026-09-04

**Authors:** Hardeep Singh, Jill I. Cameron, Michelle L. A. Nelson, Marianne Saragosa

**Affiliations:** 1 Department of Occupational Science and Occupational Therapy, Temerty Faculty of Medicine, 7938 University of Toronto, Toronto, ON, Canada; 2 Rehabilitation Sciences Institute, Temerty Faculty of Medicine, 7938 University of Toronto, Toronto, ON, Canada; 3 KITE, Toronto Rehab Institute, University Health Network, Temerty Faculty of Medicine, 7938 University of Toronto, Toronto, ON, Canada; 4 Institute of Health Policy, Management and Evaluation, Dalla Lana School of Public Health, 7938 University of Toronto, Toronto, ON, Canada; 5 Bruyère Health Research Institute, 25474 Bruyère Health, Ottawa, ON, Canada; 6 Science of Care Institute, 518775 Sinai Health, Toronto, ON, Canada

**Keywords:** stroke, stroke services, qualitative, clinicians, healthcare delivery

## Abstract

**Background:**

Best-practice recommendations for stroke care are available worldwide, yet there are challenges implementing these guidelines, contributing to gaps in stroke care. Clinicians delivering stroke services can inform improvements in stroke care delivery and outcomes.

**Objectives:**

We aimed to conduct an in-depth examination of clinicians’ perspectives on current gaps in stroke care throughout the entire care continuum, with a specific focus on the context of Ontario, Canada.

**Design:**

This is a qualitative interpretive descriptive study.

**Methods:**

Semi-structured interviews and focus groups were conducted with licensed clinicians who had experience providing stroke care in hospital or community settings in Ontario. The interviews were audio-recorded and transcribed verbatim. Analysis followed Thorne’s interpretive description analysis techniques.

**Results:**

Fourteen clinicians from five organizations participated in this study. Clinicians were occupational therapists (n=4), speech-language pathologists (n=5), physiotherapists (n=2), nurses (n=2), and a stroke coordinator (n=1). Clinicians identified the following four overarching gaps in patient- and family-centred stroke care: delays in recognition and response to stroke symptoms (theme 1), challenges in delivering and retaining stroke education (theme 2), variation in stroke care resources, quality, and rehabilitation access (theme 3), and gaps in continuity of care across the stroke care continuum (theme 4).

**Conclusion:**

The findings from this study offer critical insights into current gaps in stroke care. Addressing these patient- and family-centred gaps may improve the quality of stroke care and outcomes for patients and families.

## Introduction

A stroke requires immediate and often ongoing care due to wide-ranging effects on physical and cognitive function, and emotional well-being.^1-4^ To improve outcomes after stroke, high-quality, integrated stroke care is needed throughout the care continuum, including prevention, acute management, rehabilitation and return to community living.^5-8^ In this paper, stroke care refers to a broad range of interventions, services, and resources in hospital and/or community settings for individuals living with stroke and their families to support recovery, rehabilitation, and reintegration into the community following stroke.^8,9^

Despite advances in stroke care and the availability of stroke best-practice recommendations worldwide,^10-13^ research has identified many challenges in their implementation.^14-17^ Possibly due to the challenges implementating stroke best practices,^16,18^ gaps in stroke care, such as poor communication and information-sharing between clinicians and patients and families, a lack of comprehensive functional assessments and unmet needs, continue to be reported by patients, caregivers and clinicians in both hospital and community-based stroke care.^19-21^ Previous studies have also shown that individuals who have experienced a stroke often report unmet needs while in the hospital^22,23^ and after hospital discharge.^19,24-29^ For example, a 2019 study conducted in Canada found that individuals living with stroke felt their recovery was negatively impacted by limitations within the healthcare systems, which resulted in several unmet rehabilitation needs stemming from inequitable resource access, limited recovery options, unsupportive conversations with clinicians, and cancelled therapy sessions due to the unavailability of rehabilitation staff.^30^ A 2018 study found that individuals with stroke reported receiving “insufficient and fragmented” information, and experienced challenges accessing rehabilitation services.^23^ Fragmented care and service delivery post-stroke hinder effective care, can lead to feelings of disempowerment during rehabilitation, and contribute to unmet needs of individuals living with stroke and their families.^30-32^

The organization, funding, and delivery of stroke care systems vary across the world, with substantial differences between countries and even within regions of the same country.^33-35^ For instance, Canada’s universal healthcare system is publicly funded through taxes and has established national standards for basic coverage for insured health services.^35^ Each province and territory receives funding to administer these services, determine which services are deemed medically necessary, and oversee eligibility and the delivery of care.^35^ In Canada, while stroke best-practice recommendations are national,^12,36^ in Ontario, there is a provincial strategy outlining priorities and regionally organized stroke care delivery.^37,38^ The Ontario Stroke System, an integrated regional stroke care delivery system that aims to provide access to timely stroke care in Ontario regions, was introduced in 2000 and fully implemented in 2005.^37-40^ Ontario has 11 regional stroke systems, each with a Regional Stroke Centre and/or smaller stroke districts for larger geographical areas, that provide specialized stroke care across the full care continuum, including acute treatment, inpatient and outpatient rehabilitation, home and community care, and primary care follow-up.^38^ Despite the implementation of Canadian Stroke Best Practices^41^ and regional stroke systems in Ontario,^37,39,40^ disparities in access to stroke care and stroke care quality have been noted (e.g., access to stroke care discrepancies between urban and rural areas).^42-46^ For instance, the 2020 Ontario Stroke Report highlighted that only 31% of individuals who experienced a stroke accessed inpatient rehabilitation, which often did not meet the recommended duration of optimal therapy, and only 34% received home-based rehabilitation.^45^ As such, quality improvement of the delivery of hospital and community-based stroke care in Ontario has been recommended.^45^ Moreover, many studies continue to report unmet patient experiences and outcomes in hospital and community-based stroke care in Ontario,^19,29,47,48^ highlighting the need to further improve Ontario’s stroke care system. For example, a 2019 study by Vyas and colleagues found a reduction in unmet healthcare needs among those living with stroke compared with before 2006; however, after considering temporal trends, they did not find a link between the implementation of the integrated stroke system and unmet healthcare needs.^29^ It is important to capture clinicians’ perspectives to better understand current stroke care practices, including gaps in stroke care, as they play a central role in delivering stroke care.^9,14,15,31,49-55^ Engaging clinicians in research can help identify priorities to improve healthcare delivery and outcomes.^14,56^ However, few recent qualitative studies have examined clinicians’ perspectives on current gaps in stroke care in Ontario.

### Objective

To conduct an in-depth examination of clinicians’ perspectives on current gaps in stroke care throughout the entire care continuum, focusing specifically on the context of Ontario, Canada.

## Methods

### Design

A qualitative, interpretive descriptive^57^ study was conducted to generate insights that can inform future research. Ethics approval was obtained from the University of Toronto Research Ethics Board (REB#48577, May 23, 2025). As per our approved protocol, all participants provided verbal informed consent. The Consolidated Criteria for Reporting Qualitative Research checklist^58^ was used to enhance the comprehensiveness of our reporting (supplementary material 2).

### Setting of Study

This study was conducted in Ontario, Canada.

### Characteristics of Participants (Eligibility and Recruitment)

A purposive sample approach was used to identify licensed clinicians with experience providing stroke care in hospital or community settings in Canada. Individuals who did not work in Canada, were not licensed clinicians, and did not have any current or past experience (of any length) in stroke care were excluded. Participants were recruited through both personal networks and snowball sampling. For the latter, this involved asking individuals who met the inclusion criteria to share the study information with their networks.

### Data Collection

Before the interview or focus group, all participants completed an electronic demographic questionnaire. If more than one participant from the same institution was interested, they could choose to participate in a focus group or an individual interview. The focus groups and interviews were conducted between June 2025 and August 2025 and explored clinicians’ perspectives on current gaps, challenges, and priorities in stroke care and research (see supplementary file 1 for interview guide). Participants received a $50 honorarium in the form of an electronic gift card after the interview or focus group. The focus groups and interviews were conducted and audio-recorded on Zoom by HS, a woman Assistant Professor with occupational therapy training and experience in stroke rehabilitation and qualitative research. Participants were informed of the study’s purpose and the researchers’ goal in addressing the study objectives.

### Data Analysis

The data were analyzed following Thorne’s interpretive description analysis techniques.^57,59^ HS read all transcripts to immerse herself in the data. HS noted key phrases that were memorable, contrasting, or attention-grabbing, and revisited them during analysis to ensure they did not influence the interpretation of the rest of the data. Following this analytic stage, data were sorted into a coding framework containing broad codes that shared similar properties, recurred, and represented meaningful insights (e.g., ‘education gap’, ‘continuity of care’, and ‘resource issue’; analysis stage 2). Broad-based coding enabled the researchers to examine the data from multiple angles and explore various ways to represent it. MS reviewed two focus group transcripts representing eight participant perspectives. Then, HS and MS met to discuss initial codes and how to group them into themes to best represent the data (analysis stage 3), and any disagreements were resolved through discussion. Finally, HS created tables to understand, analyze and report the data from different angles (analysis stage 4); JC and MLAN (senior researchers) reviewed the audit trail from all analytic stages, which documented coding decisions and emerging insights. After this, JC, MLAN and HS met to discuss the codes, analytic insights and any differences in interpretations. Through this discussion, the analytic interpretations were refined, moving beyond mere descriptive accounts.

### Reflexivity

Our study, including data collection and analytic interpretations, was shaped by the research team’s training and experience. HS and MS are early career researchers with qualitative research expertise, interests in health services, and clinical backgrounds in occupational therapy and nursing, respectively. HS also has clinical and research experience in stroke care. JC and MLAN are senior researchers with expertise in qualitative stroke research and have contributed to the Canadian Stroke Best Practice Recommendations. Additionally, a participant in one of the focus groups, which was co-facilitated by MS, a woman with a PhD and qualitative research experience as an Embedded Nurse Scientist, was known to HS in a professional capacity prior to this study, which may have influenced their interactions and responses during data collection.

## Results

Two focus groups (focus group 1 with five participants (C1-C5) and focus group 2 with three participants (C9-C11)), and six individual interviews were conducted; total n=14. Table 1 presents details about the participants. All participants in this study were female and identified as women. Participants had a range of experience in stroke care from less than one year to 33 years. Participants worked across five types of organizations, including hospital-based inpatient or outpatient care (suburban), community (suburban and rural) and private care (suburban). Participants included occupational therapists (n=4), speech-language pathologists (n=5), physiotherapists (n=2), nurses (n=2), and a stroke coordinator (n=1).

**Table 1.** Clinicians’ Demographics and Practice Settings

Code	Age range (years)	Role	Description of practice setting
C1	51-60	Occupational Therapist	Hospital-based outpatient neurorehabilitation program
C2	41-50	Registered Nurse	Hospital-based outpatient neurorehabilitation program
C3	41-50	Registered Nurse	Hospital emergency department
C4	18-30	Speech-Language Pathologist	Hospital-based inpatient acute care and outpatient rehabilitation program
C5	18-30	Stroke Coordinator	Hospital-based inpatient acute care and outpatient stroke rehabilitation program
C6	31-40	Speech-Language Pathologist	Hospital-based inpatient acute inpatient care, integrated stroke unit and community-based private practice
C7	31-40	Speech-Language Pathologist	Hospital-based acute inpatient medicine unit
C8	41-50	Speech-Language Pathologist	Hospital-based outpatient rehabilitation
C9	51-60	Physiotherapist	Hospital-based integrated inpatient stroke unit
C10	31-40	Occupational Therapist	Hospital-based integrated inpatient stroke unit
C11	51-60	Occupational Therapist	Hospital-based integrated inpatient stroke unit
C12	31-40	Occupational Therapist	Hospital-based inpatient stroke rehabilitation and community-based home care
C13	31-40	Speech-Language Pathologist	Hospital-based inpatient rehabilitation
C14	51-60	Physiotherapist	Community-based home care

Four gaps in patient- and family-centred stroke care were developed through our analysis and interpretation of data collected from the interviews and focus groups: delays in recognition and response to stroke symptoms (theme 1), challenges in delivering and retaining stroke education (theme 2), stroke care system and delivery (theme 3), and continuity of care across the stroke care continuum (theme 4). Based on participants’ descriptions, our team interpreted these gaps as caused by a combination of factors at the patient-level (e.g., readiness for education), provider-level (e.g., knowledge of optimal stroke care), and systems-level (e.g., lack of staff and resources). The gaps in stroke care are summarized in Table 2, which highlights clinician-identified areas where patients and caregivers require additional support. Quotations in the results section are sourced to either Focus group 1, Focus group 2, or to individual clinicians if they are from an individual interview.

**Table 2.** Clinician-Identified Gaps in Current Patient and Caregiver Stroke Support

**Theme 1: Delays in recognition and response to stroke symptoms**	
Lack of recognition of and timely response to symptoms	Quote 1: “The education piece is missing, that they are not coming back to be seen by the doctor even after they’ve seen a symptom...so many people just walk in there like oh, I think I maybe have a stroke, and don’t understand that therapeutic window…There’s a 3 to 4 hour window…it’s a lot of times a surprise to our patients…Sounds like a long time. It’s a very short period of time you’re trying to make that decision. Do I go? Do I not go?” (focus group 1). Quote 2: “But in case I explain all that to them, and they’re like, I never knew any of that that happened, or that they’re even aware that there’s a timeframe, even they’re like, Wow, really I didn’t. Nobody told me that. It’s, it’s, amazing that nobody’s ever heard that” (focus group 1).
**Theme 2: Challenges in delivering and retaining stroke education**	
Education timing and retention	Quote 3: “How do you ensure that the information gets absorbed? And I’m sure different people are ready at different points…But I find, like in the outpatient and like in the community, is where it’s been most beneficial, because, even though we need to give the education in the acute care piece, there’s so much going on that it doesn’t always get retained, and we can say as much as we want, and like as many times as we want. But it’s not the main focus for somebody” (focus group 1). Quote 4: “Pretty much at every single point across the continuum, and everybody’s ready at different points. And so, you know, we have these care pathways in the hospital. Let’s say somebody’s had a hip replacement. There’s a care pathway on day one. They do this on day 2. They do this, and it’s a checklist to make sure all these things are addressed” (focus group 1).
Medication education	Quote 5: “Why [medication is] important to take. What is this medicine for? This is for your blood pressure. ‘I didn’t know that. I just thought it was something like a vitamin or something. Nobody told me what it's for.’ Or why you’re on blood thinners. Why, you’re on anticoagulants. The risks associated with anticoagulants as well, and when patients come in for those symptoms that happen” (focus group 1).
Caregiver education	Quote 6: “We’re seeing a lot of younger strokes and so...let’s say, disabilities and their children understanding it, their family understanding it, friends, etc., that if they share that has become really exhausting, trying to explain themselves for something that they don’t even fully understand themselves” (focus group 1).
Aphasia education	Quote 7: “From a communication perspective, I give a lot of education about aphasia, and I give a lot of education about how family and caregivers can empower a patient with aphasia to still communicate. But oftentimes I find, when they are discharged home, just because it’s faster and easier, family often still speak for them, and they don’t get to be a part of, you know, their care conversations. Some families are good about it, but I would say majority aren’t” (C6).
**Theme 3: Variation in stroke care resources, quality and rehabilitation access**	
Gaps in service allocation and staffing	Quote 8: “Depending on which region they fall under, what one offers can be really different” (C13). Quote 9: “Depends on the organization. Sometimes they have different rules like you can only be eligible 6 to 12 months since your stroke…it depends on the program...there’s a lot of inequalities based on where you are in the city” (C6)”
More time and resources for rehabilitation (initial stages)	Quote 10: “There could be more support available, especially in the initial phase of stroke, if they had more funding and research put into that initial first very crucial period. I think it would make such a huge impact on people’s recovery. Like the rehab itself, if there were more resources available, more funding available for them to maybe stay longer on the unit or get longer therapy duration” (C13).
Stroke care for people with cognitive and communication challenges	Quote 11: “[it] is frustrating…somebody with a cognitive impairment can be equally impaired and sometimes more impaired than somebody with a motor impairment, and they don’t get the same level of rehab…as opposed to getting sent to inpatient rehab where they have more time, more detailed testing, they end up getting sent home, and sometimes they get referred to us, and sometimes they don’t, because these they go to the doctor, and they can smile, and they can say yes to certain questions” (focus group 1). Quote 12: “I feel that that’s an area that gets missed quite a bit…with the cognitive communication…they can have like a simple conversation. But then, when you actually try to like probe into it, there’s some cognitive communication like issues that, like you would, they would benefit from therapy, and then it kind of just gets brushed over” (focus group 1).
**Theme 4: Gaps in continuity of care across the stroke care continuum**	
Poor information transfer across hospital and community settings	Quote 13: “For like speech-language pathologist, like for our role, like a lot of times, we’ll kind of rely a lot on like the community speech-language pathologist, or like, you know, because we can refer, for, like occupational therapist, physiotherapist, speech-language pathologist, to go nursing, to go visit the patient at home after being discharged. And a lot of times. They’ll also give a lot of the education, but they don’t have access to our notes, unfortunately, too, which makes it a little hard [to know] that’s kind of what we recommended. And so if you’re really relying on the patient from patient’s family to kind of… It would be amazing to have a care pathway that goes across the continuum of care from the emergency room to acute care, and just something that’s merged right across that maybe travels with the patient because one of the things is we don’t have a social worker on our team” (focus group 1).
Lack of communication between hospital and community providers	Quote 14: “You know what the patient was like…we worked on what the next steps would be, and if they have any questions, then they can reach out to me. But yeah, I think the main challenge is when you don’t know who’s going to be seeing your patient…Because when they leave the hospital, technically like, I’m not involved in their care anymore because they’re discharged. So after the fact, usually the community providers don’t call me” (C7).
Return to work	Quote 15: “A lot of [patients] describe it as like emotional exhaustion and returning to work and stuff and having to explain themselves and kind of the different deficits that they have because of the stroke” (focus group 1). Quote 16: “These are things that, of course, I worry about. Driving, as the occupational therapist, is a big issue, and also returning to work, because those are two higher-level areas of function. And that’s where sometimes you see, the impairments arise, and the problem is, if somebody has a cognitive impairment, yet they can speak, or they look well on the outside, and they return to work. I’ve had people where they end up losing their position because the boss or their coworkers view it as incompetence as opposed to a cognitive impairment” (focus group 1). Quote 17: “Those patients of mine who’ve gone on long-term disability or, hopefully, will get approved for long-term disability before they eventually return to work. But it can be a little bit back and forth with the insurance for them to approve a claim if somebody can speak and move fairly well, but is not cognitively ready to go back to work, whatever it might be like. I have somebody at the moment. That’s an insurance adjuster. He has to be quite intact to be able to do his job right. So it’s a little bit difficult sometimes to document that in a way that the insurance will understand...partially depends…the type of impairment, but also because of the type of job…I have somebody that has a fairly mild, cognitive communication impairment, they might be okay going back to the warehouse job they had” (C8). Quote 18: “Things get missed, and then on top of that, sometimes the doctor…says they’re okay to drive or they’re okay to go back to work without all of these things being fully addressed, because it surprises me sometimes. Even when they come in on their initial assessment, I’m having a social conversation with them, and I’ll be honest, we can’t always tell that there’s a higher level, cognitive impairment unless you engage in some sort of more complex functional activity or actual testing” (focus group 1). Quote 19: “I find one of the main barriers for those patients that I see with regards to return to work, I mean, luckily we have one very sensitive assessment available to us, like The Functional Assessment of Verbal Reasoning and Executive Strategies…So at least I can test them with evidence that there is an impairment…some people do fairly well on that like, and maybe just a little bit slow, I find sometimes they’re still not completely ready, due to like cognitive fatigue…that’s like the final limiting factor to going back to work or going back to work like even graduated” (C8). Quote 20: “I have another client who is in his mid-50s...He’s a truck driver...He wants to go back to work...But now he doesn’t have a license...his main thing is, like, getting his driver’s license back so that he can go back to work. And it’s very tricky, because it’s a right-sided weakness...like, one of the motivating things that he really wants to go back to work, because he goes, I cannot… like, I pay the bills, I have teenage sons that live with me...I need to go back to work, you know, to support” (C14). Quote 21: “Patients that come and like speak to us as spokespeople, or like presenters or whatnot, like a lot of them, do say that like it was really a struggle when they returned to work, because it was like, Oh, I didn’t know that this is how it was going to be, because I had lacking awareness and whatnot, and then, like every day, I was like struggling at work to do like one thing, and then I would like collapse in bed and fall asleep, and not be able to see my child and like. I feel like return to work is a really big gap in our service delivery model for stroke right now, because we don’t have like designated return to work centers or like, yeah, vocational rehab centers that work closely, I think, with like stroke programs” (C12).
Physical, communication and functional rehabilitation programs	Quote 22: “In the States, they have a lot of like what they call it intensive or like comprehensive stroke rehab programs, where it’s like private…I think honestly, in a way, it’s kind of better in this particular case, where it’s like you can just like forego whatever like traditional care would be like here, and just do the intensive program where it’s like very, very intensive, like every single day, for like 8 hours straight and like, I think, like for months at a time. And like, it’s a package deal. Where there’s like group programming, there’s like individual programming, like…music therapy, like the traditional like occupational speech, physio, like all of that, all kind of packaged in one, which is great. But, like obviously [patients] can’t take here in Canada, because, like, there’s no funding source for that kind of care” (C12). Quote 23: “We would probably really focus on the activities of daily living or ideals just getting back to like the regular routine which could be like a combination of physical and cognition...practice going shopping or practicing banking...doing all the house chores before being able to manage all of that, if they live alone, like, you know, are they able to manage living alone? Or are there different supports available post stroke that they can receive services from?...returning to that regular lifestyle” (C13). Quote 24: “I think just with the nature of how quickly people get seen. For example, I’m not sure if you’re aware. But the wait list right now through the [Local Health Integrated Networks] LHIN is quite long. Right? So sometimes, even for, like an occupational therapy home safety assessment and as soon as possible, but then they don’t come for, you know, four weeks” (C6).
Transportation support	“More resources and more supports overall in the community...more of support, as in, let’s say that person qualifies...to go, to outpatients after I’m, you know, while I’m working with them, they qualify to go, they’re ready to go, but they have no means to get there” (C14). “They have difficulty with stairs, so how are they going to get out of the home?...Who’s going to help with transportation to the, you know, outpatient stroke rehab program? Who’s going to finance? A lot of people are on fixed income…if they don’t have resources, if they don’t have the means to do that...And I’ve seen people refuse it, decline it, because they say, like, we can’t get there” (C14).
Financial support	“Financially, they’re not capable of going to the hospital, to the outpatient program. So it’s transportation, you know, who’s going to help with transportation to, you know, outpatient stroke rehab program. Who’s going to finance? A lot of people are on fixed income...If they can’t… if they don’t have resources, if they don’t have the means to do that...And I’ve seen people refuse it, decline it, because they say, like, we can’t get there…If the major breadwinner gets a stroke… that’s a challenge” (C14). “I have another client who is in his mid-50s...He’s a truck driver...He wants to go back to work...But now he doesn’t have a license...his main thing is, like, getting his driver’s license back so that he can go back to work. And it’s very tricky, because it’s a right-sided weakness...like, one of the motivating things that he really wants to go back to work, because he goes, I cannot… like, I pay the bills, I have teenage sons that live with me...I need to go back to work, you know, to support” (C14). “I feel like the messaging a lot of people give and like what I gave when I was like an outpatient like programming for stroke, like just a year ago, or whatnot was like, sorry like you have to go private and like people who have strokes and, like, you know, are 40, and they like, had this life debilitating thing that happened to them aren’t going back to work like they’re they don’t have the finances to pay for private” (C12).
Emotional/mental health support	“Emotional, mental health support…with strokes, there is also high incidence of emotional regulation, or difficulty with emotional regulation, and depression, and so on and so forth, so… so I think that also needs to be supported…I try to also ask for support of the social worker...I always ask to, you know, for social… when I ask for a social worker, I always ask...again, you know, there is a waitlist for social workers” (C14).
Caregiving assistance	“After a while, it comes caregiver burnout...Is the frail, elderly lady going to be able to help her older husband?...fair waitlist for PSWs at home to come and help…Waitlist for personal support workers, you know, then again, you know, with PSWs, they would also need, you know, extra hours to provide respite for major caregivers” (C14). “Sometimes it was really challenging when let’s say, they live on their own, and they don’t have a family member or caregivers. Who can, you know, help them with the practices when I’m not there, because I would only visit them once or twice, like once every week, once every other week. So it wasn’t like a regular visit either” (C13).
Linguistically tailored programs	“We do have a community aphasia program that they could access if they’re able to. It’s provided virtually and in person. It’s in English only so that’s already quite limiting. I’m in [de-identified cities] Right? So, I have lots of clients that don’t speak English. They don’t feel like it’s they speak it well enough to participate in a program like that” (C8).
Lack of services in rural and remote areas	I’m doing [virtual care] for the last 3 years? 3, 4 years, 3 years at least. And for underserviced areas, is because you can’t get a therapist...sometimes even the cell services are not available...But that’s the remote work, that’s rural areas (C14)

### Theme 1: Delays in Recognition and Response to Stroke Symptoms

Clinicians reported that they often encountered patients and caregivers who either did not recognize stroke symptoms or did not respond to them in a timely manner (Table 1, quotes 1 and 2). Participants reported that both patients and caregivers often lacked awareness of stroke symptoms and appropriate responses to stroke symptoms, which clinicians perceived as contributing to delays in acute care and potentially poorer patient outcomes: “a lot of people, let’s say, are coming too late” (Focus group 1). According to participants, although stroke response and recognition campaigns, such as the Face-Arm-Speech-Time (FAST), had been widely shared in the past, their visibility diminished over time as resources and enthusiasm for knowledge dissemination were not sustained. According to clinicians, even when people noticed their symptoms, they had difficulty determining whether and when to seek medical care.

“What I see in [the emergency department is that patients and families] miss the symptoms, or they see the symptoms, but they don't do anything about it. So they come, maybe 24 hours later, or you know, after the therapeutic window is done, and then we can't really do much for them, because they pass that window” (Focus group 1).

Our analytic interpretations of this data suggest delays in recognition and response to stroke symptoms.

### Theme 2: Challenges in Delivering and Retaining Stroke Education

Clinicians identified an important gap in patient- and family-centred stroke education (Table 2, quotes 3-7), including the challenges they faced in helping patients and caregivers retain information (Table 2, quote 3). Clinicians explained that many patients and caregivers often lacked awareness of their stroke details, current and future care plan, the impact of the stroke (such as “What can I do? What can’t I do” (Focus group 1)), how to prevent another stroke, and medication education (such as “Why am I taking this? What does this test mean that I have to go, for now” (Focus group 1)). They often heard from patients and caregivers that no one explained it to them (Table 2, quote 5). Clinicians acknowledged the challenges in educating patients, as even when they provided education, patients and caregivers struggled to retain the information. Participants attributed this retention issue to the post-stroke period being emotionally overwhelming and patients and caregivers being overloaded with information.

“Some people might be getting discharged last minute and kind of like, overwhelmed already with that getting all the medical information, they're not remembering our recommendations…There's almost like a sort of like a post-traumatic stress disorder that can occur and affect some of our clients, and sometimes we're not able to even get past, you know the education piece or that sort of thing, because they're just in shock. They're dealing with what happened. There's anxiety. There are fears in the beginning, when there's a certain degree of paralysis or swallowing issues or medical issues, whatever it is, all the fears of what's to come, and then they're not ready for the education” (Focus group 1).

Our analytic interpretations of the data suggested that, despite their best efforts to deliver stroke-related education through various formats, such as booklets and verbal presentations, clinicians reported that some people still failed to retain the necessary information. As a result, clinicians grappled with questions about the most effective formats and timing for patient education. While some preferred standardized educational pathways, such as those used in surgical care, they acknowledged that such pathways and resources have not been implemented in their practice setting, and would be difficult to apply in stroke care, given the complexity of stroke.

“Okay, we have it written out for them, or like printed copies that they can look at on their own time. But how do we know that they're engaging in it, that when you're talking to them about it, they're like, oh, this is too overwhelming, like, I can't hear it right now. So it's back to the piece of it” (Focus group 1)

Clinicians also explained that language and cultural barriers further complicated their efforts to deliver stroke-related education effectively. They struggled to find high-quality resources that were translated into specific languages.

“Where I work, there's people of like various cultures and languages, so it's also hard sometimes to have material that's accessible to people who don't speak English…having, like better handouts, maybe to explain that information would be good too. I mainly have handouts that are in English. But I have so many clients who they speak like Mandarin, or Punjabi, or Hindi. So I find it's hard to find a good resource with that information, like accessible in other languages” (C7)

Even when translated resources were available, clinicians indicated they were inadequate, as patients and caregivers may “have follow-up questions” and “you would still need the interpreter to help answer those” (C7). Clinicians also described challenges in determining whether a patient had a cognitive or communication challenge that limited their function. Clinicians in a focus group noted,

“If a person can walk and speak, if they openly on the outside look fine, they get discharged home, and many times the cognitive function and visual perceptual, the sensory does not get properly addressed” (Focus group 1).

### Theme 3: Variation in Stroke Care Resources, Quality, and Rehabilitation Access

Our analytic interpretations of the data (e.g., quotes 8-12) suggested that some clinicians experienced unease working in dedicated stroke units that lacked proper stroke accreditation (Accreditation Canada’s Stroke Distinction) as they believed these sites had lower quality of care due to limited access to specialized staff and treatments, such as speech-language and physiatry services and treatments (e.g., Botox for spasticity management). Clinicians were concerned about whether their organization was delivering optimal stroke care, as one clinician remarked, “if you are…in the city next door in [de-identified] and you have a stroke, yeah, you can get Botox…15 minutes away” (Focus group 2).

Clinicians also noted that eligibility criteria for stroke rehabilitation programs and their availability varied widely by location and changed frequently, leading to service reductions occurring over time. As one clinician stated, “Some equipment is more available, and practices are different in different regions” (C14), and that “there’s a lot of inequalities based on where you are in the city” (C6). Another clinician who was with their organization for about 18 years shared, “it gets reviewed…about every 5 years…gets reorganized like restructured…we used to do 60 minutes, now it’s 45” (C8). Consequently, clinicians desired more resources and a longer period of time to deliver therapy, particularly during the initial phase of stroke.

Another issue identified by clinicians was that inequities in care were present for patients with invisible disabilities, such as cognitive or communication impairments. Individuals with cognitive or communication impairments were less likely to receive the same level of rehabilitation as individuals with motor impairments. While clinicians noted significant gaps in the current stroke care system and delivery due to limited staff, resources, and high service demand, they were optimistic that small changes could be implemented to improve it.

“There's a lot of gaps I think we see on our end. There are a lot of gaps, and not to blame, there's a ton of demands in emergency and acute care that it's not sustainable…You don't have the staff or the resources to make everything perfect. But again…there's little things that we can, you know, change along the way” (Focus group 1).

### Theme 4: Gaps in Continuity of Care Across the Stroke Care Continuum

Clinicians described persistent challenges in maintaining care continuity during the transition from the hospital to community care. Based on our analytic interpretations of this data (e.g., Table 2, quotes 13-34), we identified several areas in which participants’ descriptions suggested unmet service needs and opportunities for improvement (e.g., a higher quality and/or quantity of services): 1) return to work, 2) physical, communication and functional rehabilitation programs, 3) transportation (e.g., to and from outpatient stroke rehabilitation and other appointments), 4) financial supports, 5) emotional and mental health, 6) caregiving, 7) linguistically tailored programs and 8) services in rural and remote areas. Based on our analysis, two key factors appeared to cause continuity of care gaps: 1) poor information transfer between hospital and community care settings and 2) lack of service availability in the community. Clinicians explained that poor information transfer prevented seamless transitions across care settings. For example, a speech-language pathologist explained that a lack of communication with the community provider and, at times, the patient’s notes not being available to the community provider hindered effective collaboration and negatively impacted patient care. One clinician expressed a desire for a more “seamlessness between the hospital and the community” (C7), and another suggested an integrated care pathway spanning from emergency and acute care to community care. They emphasized a need for “a care pathway that goes across the continuum of care from emergency room to acute care, and just something that’s merged right across that maybe travels with the patient.”

Clinicians indicated that a lack of service availability in community-based stroke care may have contributed to poor continuity of care from hospital to community settings. They also expressed concerns about their ability to provide holistic, comprehensive care to people with stroke due to limited community-based stroke care beyond the acute phase.

“There's not a lot out there for chronic stroke care like, once you're kind of done, your like acute care stay, or like your inpatient bit, you're done…unless you have another stroke, and you return to the hospital” (C12).

C13 noted that the community care gaps could be alleviated with “more resources available in the community setting…more groups, groups, or, better, follow-ups.” Clinicians also noted that even when individuals living with stroke received community-based stroke care, these services were limited in terms of time and scope, despite patients having ongoing rehabilitation goals. When community services were available to patients, they also faced long wait times, which posed problems because patients’ recovery needs were rapidly evolving.

## Discussion

Using qualitative interviews and focus groups with 14 clinicians, we identified several gaps and challenges in stroke care when providing patient- and family-centred care. These gaps are evident in four key areas: delays in recognition and response to stroke symptoms (Theme 1), challenges in delivering and retaining stroke education (Theme 2), variations in stroke care resources, quality, and rehabilitation access (Theme 3), and continuity of stroke care across the care continuum (Theme 4). Many of the comments discussed by clinicians in this study reflect gaps identified in prior studies that remain pressing issues negatively impacting the quality of stroke care in Ontario and patient and family outcomes. While education gaps, rehabilitation access issues, and fragmented transitions are entirely new insights, clinicians across hospital and community contexts described how these gaps persist in Ontario despite mature regional stroke systems and established best-practice recommendations. In particular, the findings highlight the practical tension between best-practice expectations and real-world constraints, including limited resources, variable access to rehabilitation, challenges in addressing cognitive/communication impairments, and discontinuities between hospital and community care. As such, our findings also contribute to broader discussions of practice gaps in stroke care worldwide,^14,15,49,60^ highlighting the need for future research and best practices to optimize stroke care delivery given pragmatic constraints.

### Sustainable and Widespread Public Health Campaigns Are Needed to Recognize and Respond to Stroke Symptoms

In our study, clinicians observed a significant lack of knowledge about stroke among the general public, which leads to delays in treatment. Notably, clinicians identified two main issues with stroke symptom recognition and response. First, some patients and caregivers fail to recognize the symptoms of a stroke, and as a result, delay seeking medical care. In other cases, even when symptoms are recognized, patients and caregivers often still delay seeking medical care. This delay might stem from the complexity of the decision to seek help. Research has highlighted that the decision-making process at stroke onset is complex and extends beyond mere symptom recognition.^61-64^ Public awareness campaigns focused on symptom recognition, such as those using the FAST and BEFAST (Balance-Eyes-Face-Arm Speech-Time) acronyms, have demonstrated positive effects on improving stroke symptom recognition,^65,66^ but some studies have concluded that their effectiveness is limited among individuals seeking immediate medical support when a stroke actually occurs.^67,68^ Moreover, the effectiveness of these campaigns can differ by context^63,68-70^ and their exposure, reach, long-term sustainability and success are often limited by financial resources.^71,72^ Additionally, tools and resources for stroke awareness do not always account for end-users’ specific educational and cultural needs.^73-75^ Therefore, there is an urgent need for more research to expand stroke recognition and response campaigns beyond symptom recognition and to make them more targeted, far-reaching, and sustainable beyond the initial funding period.^76^ The need for more tailored and targeted stroke symptom messaging aligns with the gaps identified in stroke symptom messages used in different countries and regions across the world.^77^

### Community-Based Stroke Care Represents a Clinician-Identified Gap in Stroke Management

Our findings emphasize the challenges clinicians face in Canada’s universally funded but resource-constrained health system to ensure that patients continue to receive care in the community. Although community-based care for people living with stroke is a best practice,^12,19,36,78^ individuals living in Canada with stroke have reported that current community programs are not effectively meeting their needs.^19^ This inadequacy may stem from a lack of programs in the community, as well as available programs not being tailored to address the diverse stroke recovery needs of those living with stroke in the community.^14,15,79^ This is an important service gap that needs to be addressed, as most individuals with stroke are discharged directly home after an acute care or inpatient rehabilitation stay with limited stroke services.^80,81^ Referral rates to outpatient rehabilitation services remain low (59%), and not all those who are referred actually receive services, particularly those who are older and score lower on functional assessments at discharge, and thus may be in greater need of ongoing rehabilitation.^81^ People with stroke and their caregivers report several challenges with accessing community-based stroke care, such as inconsistent referral mechanisms, limited awareness about and geographical access to community services, and unavailability of community programs tailored to their needs.^14,15,19^ This highlights the urgent need to reform current community-based stroke care to optimize rehabilitation and recovery, and to align with Canadian stroke best practices.^12,36,78^ Our study highlights key areas identified by clinicians that would benefit from further community-based interventions.

### Challenges in Maintaining Continuity of Stroke Care During Transitions

Improving the quality of care transitions is crucial because it can directly impact patient and family outcomes, including coping, readmissions, and functional recovery.^82^ However, clinicians have reported fragmented communication and information exchange among hospital- and community-based clinicians, patients, and caregivers during these transitions, which do not align with recommended best practices for integrated and coordinated stroke care.^78^ This finding reflects prior literature indicating that care transitions are a significant concern for older adults with complex health and social needs, including those related to stroke.^83^ This clinician-reported challenge aligns with patient perspectives identified in a quality improvement project in northeastern Ontario, where patients highlighted the need for improved post-discharge education and preparedness.^84^ Although there are innovative stroke transitional care interventions that have been shown to improve patient outcomes (e.g., physical functioning and stroke self-management) and patient experience,^71,79^ several system-level challenges to implementing these interventions persist in Ontario, including jurisdictional complexity and funding models that do not support integrated care.^83^ It is also important to recognize that gaps in care transitions are more prevalent in underserved and racialized populations.^85^ Given Ontario’s culturally diverse population, with 34.3% of Ontario residents identified as belonging to a racialized (“visible minority”) group,^86^ targeted care transition interventions may be necessary to address the challenges highlighted in this study and improve patient and family experiences and outcomes.

### Optimizing Stroke-Related Education

According to the Stroke Best Practices, patient education is considered an “integral part of stroke rehabilitation care.”^12^ It is important for enhancing the understanding of health, decision-making, health management, and caregivers’ knowledge and skills.^87,88^ However, clinicians continue to struggle with the optimal timing and delivery format of this education. While frameworks, such as Timing It Right, provide guidance on stroke education timing and delivery,^89^ meeting the diverse needs of individuals with stroke and caregivers can be challenging because of the complexity of stroke, given differences in causes, symptoms, and treatment.^87,90^ For example, past studies revealed that individuals living with stroke and caregivers, while focused on information about stroke during the acute phase, desired information on stroke management and prevention about six months later.^87,91,92^ Findings from prior literature, as well as our study, suggest that changes over time along the illness trajectory and the optimal timing of effective stroke education warrant further consideration.

In our study, we found that for some patients and caregivers, the educational materials clinicians commonly use, such as booklets, were ineffective and often left unopened.^87,93^ Cameron and Gignac (2007) recommend targeted, individualized approaches to education delivery within their Timing It Right framework; however, they also acknowledge the challenges of implementing these approaches given the limited time clinicians have for education.^89^ Nearly twenty years later, the systems-level issues, such as health care funding cuts and limited time for patient education, remain ongoing concerns that hinder effective delivery of education in stroke care. While new educational programs^94-96^ have been proposed for use within stroke care, their implementation requires efforts beyond individual clinicians. The implementation of caregiver support programs, aimed at providing education, training, and assistance to caregivers within Ontario’s regional stroke systems, has been previously explored as a way to enhance both patient and caregiver education.^94^ However, according to the perspectives of Ontario Stroke System knowledge users, implementing such a novel educational program (i.e., a caregiver support program) into stroke care requires addressing structural factors, such as personnel requirements and workflow considerations, including how these programs will integrate with existing processes.^94^

### Geographic Disparities in Stroke Care Access and Quality

Despite Canada having universal health care,^97^ clinicians in our study expressed concerns about disparities in access to and quality of stroke care across different geographic regions. These disparities are quite concerning to clinicians and should be addressed, as they believe these unfairly impact patient experiences and outcomes. For example, clinicians in our study observed variation between organizations in patient eligibility criteria for rehabilitation programs and in the equipment available. In addition, some organizations lacked specialized staff in their stroke units (e.g., speech-language and physiatry services) and access to treatments (e.g., Botox for spasticity management), which limited their ability to refer patients to these services. Our findings align with a pan-provincial survey of stroke units in Ontario, which highlights regional variations in access to stroke units and the need for specialized stroke training for clinicians, with half of the units failing to meet all the criteria outlined in the Canadian Best Practice Recommendations for Stroke Care.^42^ Taghdiri and colleagues (2023) identified similar geographic disparities in Ontario, specifically related to “thrombolysis or thrombectomy for acute ischemic stroke.”^98^ From the clinicians’ perspectives, the availability of resources within their organization and in the surrounding community significantly influences their decisions about stroke care provision, and may contribute to patients “falling through the cracks.”^51^

### Strengths and Limitations

Our study included clinicians from hospital and community-based settings, with varying levels of experience in stroke care, ranging from less than 1 to 33 years. The analysis involved multiple researchers, including two senior researchers with extensive stroke research experience and who have contributed to the Canadian Stroke Best Practices. However, it is important to note that most of our sample worked in a hospital setting. Another limitation of this study is its cross-sectional design, which limits understanding of temporal changes and the factors influencing stroke care delivery. Our study findings aligned with and extended previous literature, including some studies conducted by our research team. However, this study could be repeated in the future to capture current gaps in stroke care from a national perspective. Another limitation is that the interview questions were not pilot tested, which may have affected their clarity. However, as the interviews were semi-structured, the interview questions were adapted for clarity and to probe participant responses. Additionally, one participant was known to the researcher, which may have influenced their interactions during data collection. Finally, our study reports only the perspectives of clinicians and excludes the voices of non-clinicians, such as patients, their families, and other relevant knowledge users. Future studies could include the perspectives of patients, caregivers, and health system leaders to complement this study’s findings related to pressing priorities in current stroke care delivery. Future research should also identify more effective implementation strategies that better align with the realities of clinical practice.^53,55^

## Conclusion

This qualitative study provides an in-depth description of current clinician-identified gaps in hospital- and community-based stroke care in Ontario. Our findings highlight multiple areas within Ontario’s stroke care continuum that clinicians perceive as needing strengthening. Although there is a strong commitment to high-quality, evidence-informed stroke care in Canada, clinicians in our study have reported difficulty achieving this goal. Future research is needed to determine how to address these gaps within the pragmatic constraints of stroke systems.

## Supplemental Material

Supplemental Material - Identifying Clinician Perspectives on Current Stroke Care Gaps Across the Care Continuum: An Ontario, Canada-Focused Study Supplemental Material for Identifying Clinician Perspectives on Current Stroke Care Gaps Across the Care Continuum: An Ontario, Canada-Focused Study by Hardeep Singh, Jill I. Cameron, Michelle L. A. Nelson, and Marianne Saragosa in Health Services Insights.

Supplemental Material - Identifying Clinician Perspectives on Current Stroke Care Gaps Across the Care Continuum: An Ontario, Canada-Focused Study Supplemental Material for Identifying Clinician Perspectives on Current Stroke Care Gaps Across the Care Continuum: An Ontario, Canada-Focused Study by Hardeep Singh, Jill I. Cameron, Michelle L. A. Nelson, and Marianne Saragosa in Health Services Insights.

## Data Availability

The datasets generated and/or analysed during the current study are not publicly available due ethical restrictions but are available from the corresponding author on reasonable request.*
