# Supplementary material for: Identifying Clinician Perspectives on Current Stroke Care Gaps Across the Care Continuum: An Ontario, Canada-Focused Study
Source: Health Serv Insights. 2026 Sep 4;19:11786329261486701. doi: 10.1177/11786329261486701 (PMC13547806; doi:10.1177/11786329261486701)
Supplement: Supplementary file 1 [file sj-pdf-1-his-10.1177_11786329261486701.pdf]

1. What are the current gaps in stroke care clinical practice you see? Based on your clinical experience, what stroke care challenges have your patients, their families, and caregivers faced? Consider policy-, hospital-, patient-, service- or community-level challenges.
    - How can these be addressed?
    - What should researchers look at to help clinicians improve stroke care delivery?
2. Based on your clinical experience, what stroke care-related challenges have you faced that may benefit from additional research?
    - Given these challenges, which might be essential to study further and why?
3. How does research, which could mean evidence from guidelines, papers, or data from reports, inform your work as a clinician in stroke care?
4. How can stroke research help improve patient outcomes (e.g., function, quality of life)? patient care experiences (e.g., experience in the hospital, during transitions)?
5. Ideally, how would you like to be involved in stroke research (if at all) (i.e., generating research ideas or questions, designing the study, contributing to the data, or hearing about the research and integrating findings into practice)?
    - How do you learn about opportunities to participate in research?
    - Can you please share past experiences of being involved in a research study? E.g., role (participant, collaborator)
    - What worked well? What did not work well? Any suggestions for other researchers to consider?
    - What should researchers know/consider when doing research with stroke care providers and clinicians? How can researchers ensure meaningful involvement? Probe about person-related factors- communication preferences, context-scheduling, timing, etc;
6. Based on your experience of being involved in research, what kind of factors need to be considered when thinking about doing research?
    - organizational culture (e.g., leadership support), capacity (e.g. time, understanding of practice based research, structures in place to be involved in research) to do research value – is there value for you being involved in research
7. How do you learn about new research findings related to your work?
    - What makes learning about new research findings related to your work hard?
    - What makes learning about new research findings related to your work easy?
8. How can researchers effectively share research findings with stroke care providers and administrators?
9. In your experience, what prevents research findings from changing clinical practice?
    - E.g., attitudes, administrator/leadership support, time constraints?
10. In your experience, what has helped research findings create change in clinical practice?
